# Action Plan to enhance self-management and early detection of exacerbations in COPD patients; a multicenter RCT

**DOI:** 10.1186/1471-2466-9-52

**Published:** 2009-12-29

**Authors:** Jaap CA Trappenburg, Lieselotte Koevoets, Gerdien H de Weert-van Oene, Evelyn M Monninkhof, Jean Bourbeau, Thierry Troosters, Theo JM Verheij, Jan-Willem J Lammers, Augustinus JP Schrijvers

**Affiliations:** 1 Julius Center for Health Sciences and Primary Care, University Medical Center Utrecht, Heidelberglaan 100, 3584 CX Utrecht, The Netherlands; 2, Respiratory Epidemiology and Clinical Research Unit, Montreal Chest Institute, McGill University, 3650 St. Urbain Street, Montreal, Canada; 3, Department of Rehabilitation and Respiratory Division, University Hospital Gasthuisberg, Katholieke Universiteit Leuven, B-3000 Leuven, Belgium; 4 Department of Respiratory Medicine, University Medical Center Utrecht, Heidelberglaan 100, 3584 CX Utrecht, The Netherlands

## Abstract

**Background:**

Early detection of exacerbations by COPD patients initiating prompt interventions has shown to be clinically relevant. Until now, research failed to identify the effectiveness of a written individualized Action Plan (AP) to achieve this.

**Methods/Design:**

The current multicenter, single-blind RCT with a follow-up period of 6 months, evaluates the hypothesis that individualized AP's reduce exacerbation recovery time. Patients are included from regular respiratory nurse clinics and allocated to either usual care or the AP intervention. The AP provides individualized treatment prescriptions (pharmaceutical and non-pharmaceutical) related to a color coded symptom status (reinforcement at 1 and 4 months). Although usually not possible in self-management trials, we ensured blinding of patients, using a modified informed consent procedure in which patients give consent to postponed information. Exacerbations in both study arms are defined using the Anthonisen symptom diary-card algorithm. The Clinical COPD Questionnaire (CCQ) is assessed every 3-days. CCQ-recovery time of an exacerbation is the primary study outcome. Additionally, healthcare utilization, anxiety, depression, treatment delay, and self-efficacy are assessed at baseline and 6 months. We aim at including 245 COPD patients from 7 hospitals and 5 general practices to capture the a-priori sample size of at least 73 exacerbations per study arm.

**Discussion:**

This RCT identifies if an AP is an effective component of self-management in patients with COPD and clearly differentiates from existing studies in its design, outcome measures and generalizability of the results considering that the study is carried out in multiple sites including general practices.

**Trial Registration:**

NCT00879281

## Background

Chronic obstructive pulmonary disease (COPD) is characterized by airflow limitation that is not fully reversible. This airflow limitation is usually progressive and associated with an abnormal response to noxious particles or gases [1]. COPD is a major cause of morbidity and mortality throughout the world[2]. Its stable state is interrupted by periods of worsening symptoms which vary in severity and frequency both during the course of a patient's illness and between patients. Depending on aetiology and severity, these periods may be referred to as exacerbations. Exacerbations are important because of their impact on morbidity and mortality. They may hasten disease progression by accelerating the decline in lung function [3,4] and have a significant effect on quality of life [5,6]. Adjusted for disease severity, patients with exacerbations show higher mortality rates than patients without exacerbations [7,8].

According to international guidelines, exacerbations should be treated with inhaled bronchodilators (particularly inhaled beta2-agonists with or without anticholinergics) and oral corticosteroids. Patients experiencing COPD exacerbations with clinical signs of airway infection (e.g., increased sputum purulence) may benefit from antibiotic treatment [1]. Not all exacerbations are captured by reliance on healthcare contacts. Previous studies have shown that less than 50% of exacerbations will not be reported to the healthcare providers and subsequently do not receive the correct treatment [9-11]. It is debatable whether unreported exacerbations are sufficiently mild that they can be disregarded. More than that, patient recognition of exacerbations and prompt therapy have shown to improve exacerbation outcomes [12]. In addition, patients who refrain from seeking treatment for an exacerbation or have lower self-management capacity show higher hospitalization rates compared to those who seek early treatment from physicians or have better self-management capacity [13]. This underlines the relevance of improving self-management skills to enhance early detection and taking early and appropriate actions by patients in exacerbation episodes.

Until now, little data is available on which methods are effective in enhancing self-management associated with exacerbations. A potential effective method to help patients to recognize and anticipate on the early symptoms of an exacerbation, is an 'action plan' (AP). APs encourage patients to identify daily variations in symptoms and when needed, take the appropriate actions, i.e. change medication regime, or visit a health care provider. Until recently, evidence on effectiveness of using APs is relatively moderate. A systematic Cochrane review including only three randomized controlled trials with relatively small sample size and methodological limitations showed no effects of APs on clinical COPD outcome parameters or healthcare resources [14]. Nevertheless, it was concluded that APs provide an approach to increase patients' exacerbation-related self-management strategies. However, data are still insufficient to draw conclusions in terms of clinical outcomes or healthcare utilization. These findings highlight the need for continued research in this field using high quality randomized controlled trials with adequate sample size.

This present study aims at evaluating the effectiveness of an AP as an addition to care as usual in a randomized controlled trial. We hypothesize that in the event of an exacerbation an AP aiming at early contacting healthcare providers and thus prompt intervention, leads to faster recovery in symptoms and health status in patients with COPD. Secondary outcomes will include self-management behaviour such as contact/treatment delay, self-efficacy and healthcare utilization. This article provides a detailed description of the study background, the targeted population, study methodology and subsequently compares this with previous randomized trials regarding effectiveness of APs in COPD treatment.

## Objectives

### Main objective

To evaluate effectiveness of an individualized Action Plan on health status recovery time (days), in the event of an exacerbation.

### Secondary objectives

To evaluate effectiveness of an individualized Action Plan on in the event of an exacerbation on, symptom recovery time (days), treatment delay, healthcare utilisation, health-related quality of life (HRQL), depression and anxiety and self-efficacy.

## Methods

### Design

The study is conducted as a single-blind randomised controlled trial, with a 6 months follow-up. Patients are randomly assigned to either care as usual or treatment with an individualized AP as an addition to care as usual. The randomisation procedure is performed by a respiratory nurse (RN). To conceal the assignment sequence, a central web-based service on a 1:1 basis is used. Randomisation is stratified by centre and gender.

### Blinding and ethical considerations

Fundamental bias can be introduced in randomized trials if patients cannot be masked for the allocated intervention and subjective outcomes are assessed. For obvious reasons, blinding in behavioural and self-management interventions is usually not possible. However, a modified informed consent procedure enables a single-blind design. In the present study an informed consent to postponed information procedure is used, keeping the patient unaware of the AP being the major study aim. This procedure is used to deal with serious threats to internal validity and justified since the intervention entails no risk. Using a regular full informed consent procedure and by absence of a placebo intervention, patients would be fully aware of the study purpose and treatment allocation. This awareness of patients might lead to biased results, especially because the primary outcome measures of this study are subjective (e.g. HRQoL and symptoms). This postponed informed consent procedure has shown to be a valuable solution to obtain valid assessment of subjective outcomes in a trial in which patients cannot be blinded to the intervention[15,16], also in patients with COPD [17]. In addition, in a Dutch cohort, patients did not have objections to this procedure and did not refuse participation more often[18]. Our modified informed consent procedure implies that all patients are informed about the fact that, besides the outcome assessment aiming at gaining more insight in daily symptom variations, the study has another purpose. Patients are told that they will be informed about this additional research question only after follow-up because informing during recruitment would affect study results. A letter with postponed information about the additional research question, the randomization, allocation and reasons for not informing the patient during recruitment is sent after the collection of all outcome data. Meanwhile, the medical ethical committee of the University Medical Center Utrecht has approved this procedure (08-275). Using the modified informed consent procedure in our trial, we deal with the following threats to internal validity:

#### Selection bias by attrition or dropout

Patients' preference for allocation to the treatment arm and receiving "the innovation" above care as usual might result in increased dropout in the control group, due to being dissatisfied or lack of interest[19].

#### Resentful demoralization/compensatory rivalry

Behaviour of the control group may alter as a result of the study, not due to the independent variable (intervention vs. control). In our study, the control group might be dissatisfied not receiving "the innovation", which increases the risk of biased results. Changes in outcome may only be affected due to a demoralized and perhaps less motivated control group not due to the independent variable. In addition, control patients may compensate by changing their behaviour, which might result in dilution of the intervention effect.

#### Desire to please the investigator/loyalty bias

Patients in the intervention group, who receive "the innovation", might make more favourable follow-up assessments out of loyalty to the programme's staff.

#### Contamination

By knowing that the AP aims at early recognition and action in case of changing symptoms, control patients behaviour might also be influenced by this undesirable knowledge. If subjects in the control group adopt intervention behaviour, a lack of differences between experimental and control groups may be observed (dilution of the effect).

This study has been approved by the Medical Ethical Committee (08-275) of the University Medical Centre Utrecht. Written informed consent is requested from all patients prior to participation in the study.

### Study population

Patients are selected based on the following inclusion criteria: 1) post-bronchodilator FEV_1_/FVC ratio < 70%; 2) age > 40 years; 3) smoking history of more than 20 years or 15 pack-years; 4) diagnosis of COPD as major functionally limiting disease and 5) current use of bronchodilator therapy. Exclusion criteria are: 1) primary diagnosis of asthma (onset < 35 years, ≥ 12% post-bronchodilator reversibility); 2) primary diagnosis of cardiac disease and 3) presence of disease that could either affect mortality or participation in the study (e.g. confusional states).

### Recruitment and informed consent

**Figure 1:**
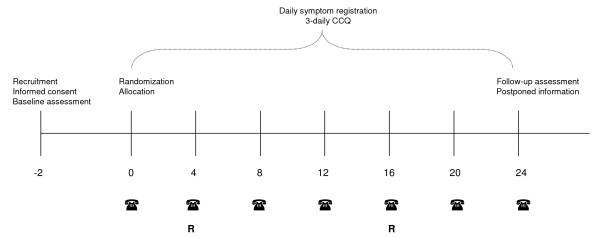
**Schematic overview of the study procedure**. Patients are recruited and informed, two weeks before randomization. R: Reinforcement of AP use by telephone, only for the intervention group.

Patients are recruited from seven regional hospitals and five general practices in the Netherlands. Inclusion takes place within a period of 5 months (winter - spring). Medical records of patients with a scheduled visit to a respiratory nurse or practice nurse (from here referred to as respiratory nurse) are screened for in- and exclusion criteria by a respiratory or family physician. Eligible candidates receive an information letter about the content of the study two weeks prior to a scheduled (regular) visit to the respiratory nurse (figure 1) including an informed consent form. One week before this visit, patients are contacted by telephone by one of the researchers to get their permission to send a baseline assessment questionnaire and to provide additional information when needed. The informed consent form and baseline questionnaire are turned in at the visit to the respiratory nurse.

### Control group

Usual care consists of both pharmaceutical and non-pharmaceutical care according to most recent evidence-based guidelines on COPD care[1]. At inclusion all patients are seen by a respiratory nurse who systematically checks and discusses the following aspects of COPD care: vaccination, optimizing medication, inhalation techniques, exercise, nutritional aspects, smoking (cessation) and exacerbation management. When needed, additional instructions or information is provided including information booklets, referral to a physiotherapist, dietician etc. To control for any co-intervention, all nurses are asked to record the different aspects discussed in this session. During follow-up, patients have normal access to their physician and respiratory nurse. No attempts are made to change the frequency of scheduled visits.

### Intervention

In addition to care as usual, patients in the intervention group receive an individualized Action Plan. The first version of the AP was developed using a combination of the Anthonisen classification of exacerbation symptoms and signs[20], and the AP used by Watson et al[21]. To optimize the AP for disease-related patient utilization and to guarantee that the AP does not conflict with evidence-based guidelines on COPD care, five experts in the field of COPD self-management (n = 3) and COPD guidelines (n = 2) were consulted using semi-structured interviews. With the resulting version of the AP a pilot study was conducted from December 2007 until May 2008. In this study, 121 (response rate 71%) COPD-patients (age 67.4 ± 10.5 years, FEV_1 _47.7 ± 18.5% pred) received an AP and were followed up for 42 days. Thirteen patients were lost to follow-up (11%). Patients reported both format and content of this AP as highly satisfactory. In this pilot study we acquired experience and understanding in patient recruitment, patient response, study procedures in self-management research, daily diary symptom assessment/analysis and patients' perception of AP use.

The final version provides a colour-coded overview of patients' stable and deteriorated symptom status related to individualized pharmaceutical and non-pharmaceutical treatment prescriptions. The backside of the AP includes three sections which can be used to provide additional (individualized or general) information on medication, exercise and nutrition. The AP is printed on A3-sized paper and folded as a brochure. Standardized instructions on how to use the AP are provided by the respiratory nurse. An information brochure is provided as well. In addition the AP is individualized by the respiratory nurse together with the patient by filling in 1) a list of important contact persons and telephone numbers; resource persons: family physician, respiratory physician and respiratory nurse; 2) stable symptom severity (individual stable/normal green zone symptom status); 3) regular medication/lifestyle prescriptions (green zone); 4) additional medication/breathing exercises and energy preservation in case of symptom increase (yellow zone, orange zone); 5) name contact person/telephone number in case of an exacerbation (orange zone)

The AP is clearly not a one-moment 'paper' intervention. After being instructed on the individualized AP content and use, the patient is instructed to bring the AP to every visit to a healthcare provider. In these follow-up visits, the AP can be changed or made complete. Within the study period, there will be two standardized reinforcement sessions by telephone at 1 and 4 months, performed by an independent respiratory nurse (figure 1). In these sessions, patients understanding and adherence concerning AP use is evaluated and when needed additional information is provided.

### Outcomes and measurements

#### Exacerbations

**Figure 2:**
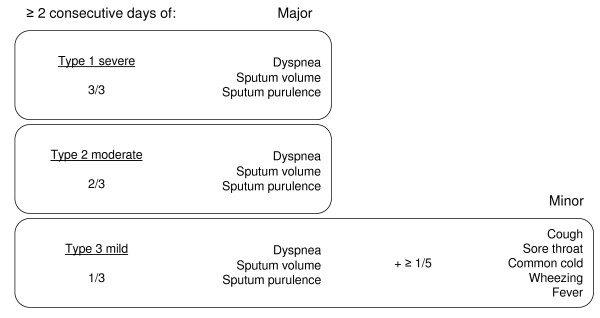
**Symptom-based exacerbation algorithm according to Anthonisen et al**.

Exacerbations are detected by daily recording of symptoms. Symptoms recorded are termed major and minor (figure 2) according to Anthonisen et al[20]. A symptom-based exacerbation is defined by the onset of two or more new or deteriorated symptoms, of which at least one major symptom, persisting for 48 hours or more. A subsequent exacerbation is defined as a new event only in the presence of at least two stable weeks. Exacerbation onset is defined by the first day on which the symptom criteria are met. Daily symptoms are binary coded and summed to give a daily symptom count. Major are scored as: normal = 0; small increase = 1; or clear increase = 2. The minor symptoms are scored 0 and 1 respectively. Adding all these scores results in a daily symptom count with a range from 0-11 points. Exacerbations are recovered when the three-day moving mean of the symptom count returns to the symptom count of days fourteen to eight prior to exacerbation onset.

#### Primary outcome

##### Health status recovery time

**Figure 3:**
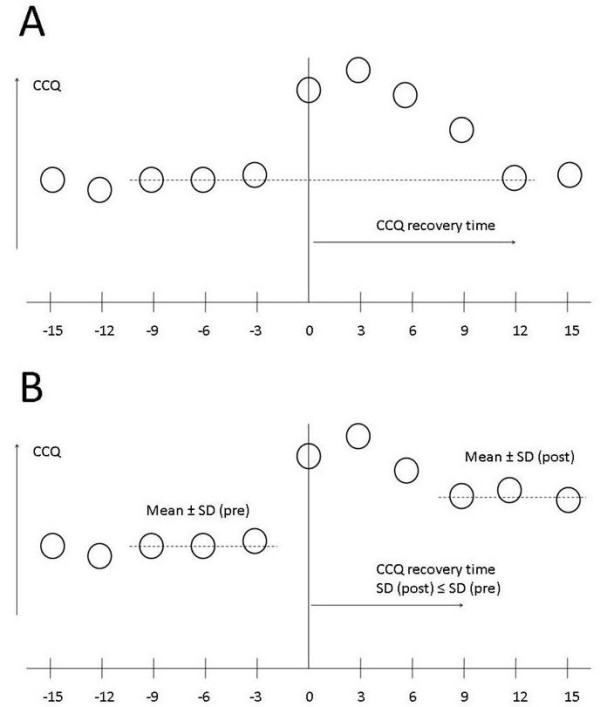
**CCQ recovery time**. Example of CCQ recovery time in a patient with full recovery (A) and a patient who does not fully recover but has a new baseline (stable) condition after 9 days = 3 units (B).

The Clinical COPD Questionnaire (CCQ) score is used to measure disease-related health status. The CCQ is a validated ten-item questionnaire, divided into three domains: symptoms, functional state and mental state[22]. In this study the 24-hour version is used, in which patients are asked to record their experiences during the last 24 hours. Recovery time of health status in the event of an exacerbation is defined by the number of CCQ units from the onset of a symptom-based exacerbation, up to the moment CCQ score is back to its pre-exacerbation average (figure 3). If a patient does not fully recover from an exacerbation, the CCQ-recovery time is defined by the onset of the exacerbation and a new stable condition, deflected by discontinuation of recovery for three consecutive units. A new stable condition is present when the standard deviation of the new baseline is less than the pre-exacerbation three units' standard deviation.

#### Secondary outcomes

##### Symptom recovery time

Symptom recovery time is calculated as the number of days from exacerbation onset for the 3-day moving average of the exacerbation daily symptom count to return to the mean symptom count on day fourteen to eight preceding exacerbation onset. The use of a 3-day moving average minimises the effect of day to day variations without biasing the results[12].

##### Health-related quality of life

The St George Respiratory Questionnaire (SGRQ) is used to measure health-related quality of life (HRQL). It is validated for measuring health impairment in COPD patients. The SGRQ consists of sixteen questions in three domains: symptoms, activity and impact. It assesses patients' perception of respiratory problems over a preceding period, ranging from one month to one year, as well as their current state[23]. Both between group differences in SGRQ scores will be assessed as well as the proportion of patients crossing the clinical relevant threshold of 4 points [24].

##### Unfavourable health status

Unfavourable health status days per patient-year is defined as the number of days on which the CCQ score is = the individual CCQ-score mean (total follow-up time) minus one standard deviation. Each patients' score is divided by their follow-up time (patient - year).

##### Anxiety and depression

The Hospital Anxiety and Depression Scale (HADS) is used to measure symptoms of anxiety and depression. Both domains consist of seven statements on emotions or emotional situations. Patients express their agreement with the statements on a scale from 0 to 3, which leads to a maximum score of 21 points for each domain. Scores between 8 and 11 per domain are suggestive of the presence of the mood disorder; scores = 11 indicate a probable presence[25].

##### Self-efficacy

Exacerbation-related self-efficacy is measured by a self-developed questionnaire, consisting of 11 items. Patients are asked to grade their confidence in their own self-management capabilities in the occurrence of an exacerbation on a 5-point Likert scale. Lower scores indicate high confidence in adequate exacerbation related self-management behaviour. No data is available on validity and responsiveness of this questionnaire, this will be addressed simultaneously to the current study.

##### Contact delay

The number of days between onset of an exacerbation and contact of a health care provider.

##### Treatment delay

Number of days between the onset of an exacerbation and initiation of antibiotics or oral corticosteroids.

##### Exacerbation rate

• Proportion of patients that experience a symptom-based exacerbation.

• Proportion of patients that experience an event-based exacerbation, i.e. the occurrence of an exacerbation for which antibiotic or corticosteroid treatment is initiated.

• Number of symptom-based exacerbations per patient year, by dividing each patients' exacerbation rate by their follow-up time.

• Number of event-based exacerbations per patient year, by dividing each patients' exacerbation rate by their follow-up time.

##### Health care utilization and cost-effectiveness

The number of respiratory-related hospital admissions, hospital days, emergency room visits and scheduled and unscheduled visits or telephone calls to both respiratory and family physicians are assessed. The proportion of patients having one or more respiratory-related hospital admissions is determined, as well as the number of days between inclusion and first hospital admission.

The perspective of the healthcare payer is adopted for the cost-effectiveness analysis (CEA). Resource use is valued in monetary terms by multiplying the units consumed by the cost per unit[26]. Two major cost categories are distinguished: program implementation and direct health care. Data on cost and outcome are brought together to estimate incremental cost-effectiveness ratios (ICERs) and subsequently calculated as costs per exacerbation-related CCQ unit prevented, per hospital admission prevented, and per health care contact prevented. Sensitivity analysis is used to test the cost-effectiveness model for methodological uncertainty and generalizability.

### Study procedures

#### Instruction of healthcare providers

All healthcare providers are well informed about the study procedures in three, 2-hour meetings with the research group. They are explicitly instructed not to talk about randomization and allocation to their patients. Written information is provided as well.

#### Baseline assessment

Baseline parameters include socio-demographic and anthropometric parameters, MRC dyspnoea scale, HADS, SGRQ, the self-efficacy questionnaire and lung function values. Medical records are checked for the presence of lung function assessment in the previous three months. If absent, spirometry is scheduled before inclusion. Post-bronchodilator Forced Expiratory Volume in one second (FEV_1_), Forced Vital Capacity (FVC) and FEV1/FVC ratio are assessed.

#### Follow-up assessment

Patients are instructed to record any increase in a predetermined list of symptoms over their chronic (stable) symptoms during the previous 24 hours. Every third day the CCQ is completed. Patients receive six diary booklets, each consisting of 30 days. After completing one of the diaries, patients are asked to send them to the investigator. Every four weeks patients are contacted by telephone to assess healthcare utilization and to evaluate proper use of the diary. At 24 weeks, HRQL, anxiety and depression, dyspnoea and self-efficacy are reassessed. After filling in the last diary and follow-up questionnaire, patients receive a postponed-information letter about the aim of the study and AP and their allocation. Additional data regarding health care consumption (exact date and content of visits and interventions) are obtained from medical records.

### Sample size

Based on two studies, a median health status recovery time around 12 (IQR 8-16) days is expected [9,27]. Unfortunately we do not know the shape of the underlying distribution. Nevertheless, a nonparametric test might require either more or fewer subjects compared to a Students' t-test, but they never require more than 15% additional subjects if 1) the number of subjects is reasonably high: (how high depends on the nature of the distribution and test, but figure at least a few dozen) and 2) the distribution of the data is not unusual (does not have infinite tails, in which case its standard deviation would be infinitely large)[28].

Hence, we adopted the general rule for computing the sample size required for a t-test. In a normal distribution the distance between the first quartile and the median is approximately 0.67 times the standard deviation of the distribution. Thus, if we assume the distribution of recovery time (median = 12, IQR = 8-16) to be normal, the mean of this distribution will be 12, with standard deviation 6. Sample size is calculated based on the smallest detectable change of one 3-day CCQ unit. Using the aforementioned 15%-rule, would mean a sample size of 63 (two tailed t-test) * 1.15 (+ 15%) = 73 exacerbations are needed in both arms to have an 80% chance of detecting a difference, using a two side alpha = 0.05.

To optimize generalizability, pre-study exacerbation rates are deliberately not part of the inclusion criteria. The number of patients needed to obtain 73 exacerbations per arm depends on the expected 6-months symptom-based exacerbation incidence. Symptom-based exacerbation incidence has shown to be at least 50% higher compared to exacerbations based on healthcare contacts [9-11]. Different prospective cohorts studies using similar symptom-based algorithms in average populations found (median or mean) exacerbation rates ranging from 2.53 (IQR 1.33-3.80)[4], 2.7 (± not-reported), 3.5 (± 2.7)[29] to 5.0 episodes (IQR, 4.0 to 7.0)[11] per patient per year. Based on these rates, the proportion of patients experiencing at least one episode is expected not to be less than 70% and subsequently is a safe boundary. Since symptom-based exacerbation recovery is the main outcome measure, dropout is defined as premature and for any cause interrupting or ending daily symptom registration, for at least 3 weeks. Data on dropout rates in similar randomized trials using daily symptom monitoring are relatively scarce. Two studies documented a dropout rate of 14% [29] and 12% [30] respectively.

Presuming a 6-months symptom-based exacerbation incidence of 70%, 15% loss to follow-up and 60% response rate this means 409 patients need to be recruited to include 282 patients having 146 exacerbations.

### Data analysis

SPSS 15.0 for Windows (SPSS, Inc., Chicago, IL) will be used for data analysis. Baseline characteristics are expressed as means and standard deviation or medians and interquartile range. All data will be analysed as intention to treat. Differences in CCQ symptom recovery time between both groups will be evaluated using Mann-Whitney *U *test. Number of exacerbations will be reported as weighted exacerbations rates (total number of exacerbations divided by the total person-time of follow up per group). This approach has shown to produce unbiased estimates of a weighted statistical approach adjusted for asymmetry in follow-up times by accounting for each patient's time spent in the trial [31,32]. Statistical significance of weighted rate ratio's are calculated using a Poisson regression model including an overdispersion parameter to account for variability in exacerbation rates between patients. Other variables will be analyzed using paired student t-test (continuous data), chi-square test (binominal data) and Mann-Whitney *U *test (ordinal data). To determine the magnitude of the treatment effect, Cohen's effect sizes are calculated for continuous variables measuring differences in pre- and post-change.

Confidence intervals of the incremental cost-effectiveness ratio's are tested using nonparametric bootstrapping (Monte - Carlo simulation) drawing 100.000 samples[33].

## Discussion

Early detection of exacerbations by COPD patients has shown to be clinically important. To achieve this, unfortunately, little data is available on which methods can enhance exacerbation-related self-management. This also applies for the potential benefits of AP interventions. Although comprehensive self-management programs have been extensively studied, effectiveness of an AP as a single self-management intervention has been hardly investigated. A Cochrane review [14] shows that only three studies evaluated benefits of an AP as a single intervention. These studies teach us that AP's enhance recognition of exacerbations and taking appropriate action measures timely, but were clearly underpowered (sample size) to find effects in the aimed outcome measures. The current study aims at the evaluation of the hypothesis that a '*written*' AP enhances early detection and prompt action and is consequently beneficial in exacerbation outcome (i.e. recovery time).

A priori methodological decisions in the current study are compared with the strengths and weaknesses from past randomized trials. The study characteristics (table 1) show that the current study has some innovative features and differentiates from the other three studies evaluating isolated effects of an AP as an addition to care as usual. Within the current study, an intentional multicenter design, targeting at inclusion from both general practices as well as outpatient clinics was chosen. This optimizes not only the external validity of the study allowing better generalization of the results but also enables to capture both patients with mild airway obstruction and the most vulnerable patients (high exacerbation-rates).

**Table 1:** Characteristics of studies evaluating isolated effects of an AP as an addition to care as usual.

	Current study	Watson	Wood-Baker	McGeoch
***Procedure***				
Recruitment	OC + GP	GP	GP	GP
Pilot-study	+	+	-	+
Intervention				
- Early contact	+	+	+	+
- Self-initiation drugs	-	+	+	+
- Reinforcement	+	-	-	-
Outcome	exa-rt	SGRQ	SGRQ	SGRQ
***Validity***				
Randomization	individual	individual	clustered	clustered
Blinding	pt + inv	-	-	-
Stratification	by centre	-	-	-
***Statistics***				
Follow-up (mths)	6	6	12	12
n		56	102	151
Powered on	Δ exa-rt = 1	Δ SGRQ = 5.7	Δ SGRQ = 4	Δ SGRQ = 4
SSC	245	124	124	160

OC, outpatient clinic; GP, general practice; exa-rt, exacerbation recovery time; SGRQ, St George respiratory questionnaire n, number of patients; SSC, sample size calculation; pt, patient; inv, investigator.

Usually, blinding in self-management or educational interventions is nearly impossible, but the so called 'modified informed consent' procedure, does enable a single-blind RCT and consequently compensates for substantial threats to internal validity. In the field of disease management research this can be regarded innovative in terms of methodology.

In the studies of Wood-Baker et al[34] and McGeoch et al[35], randomization was clustered to prevent cross-contamination of educational messages within a practice. Selection bias in cluster randomized trials may threaten the validity of the results. These studies however did not inflate their sample size to accommodate for this clustering effect [36]. We believe that in this robust study (245 patients) the impact of contamination is regulated by the modified informed consent procedure (patient not knowing that the AP is part of the study objective) and the strict instructions of RN's not to inform control patients about the existence of the AP. The remaining threat of contamination does not weigh against the disadvantage of the huge sample size needed in a clustered trial to compensate for the high inter-cluster variations in both patient (i.e socioecomic differences) and healthcare (i.e. number and content of respiratory nurse consultations) characteristics.

Although all studies used an individualized AP, aimed at early recognition of exacerbations, there are some differences between the interventions. Instead of encouraging self-initiation of antibiotics or oral corticosteroids, the AP used in the current study aims at early contact with a healthcare provider only. The choice to initiate pharmaceutical treatment is left to the physician. Moreover, we have respiratory nurses to evaluate use of the AP and provide additional information when needed. Reinforcement has shown to be essential in changing self-management behaviour, leading to better use of the intervention [37].

Finally, the current study clearly differs from the other studies in the primary outcome on which the study is powered. Aiming at evaluating effectiveness of a single component intervention (AP) necessitates being reserved on powering on general outcome measures such as hospital admissions. Therefore, the current study aims at specific and accurate assessment of effectiveness of the AP on patient-centred outcomes, in the presence of an exacerbation, since this is what the AP is developed for. In addition, a clinical relevant advantage of assessing exacerbation recovery time is that it assesses effectiveness in all exacerbation episodes and not only those followed by healthcare contacts or hospital admissions. Effectiveness in terms of decreased CCQ recovery time is equivalent to a decrease in the impact of these episodes on health status and thus highly clinical relevant from a patient perspective. Detecting an effect on recovery time of exacerbations, the primary outcome, is an essential first step before trying to prove that the intervention can increase HRQL or reduce hospital admissions. In conclusion, this RCT identifies whether an AP is an effective component of self-management in patients with COPD and clearly differentiates from existing studies in its design, outcome measures and robustness.

## Competing interests

The authors declare that they have no competing interests.

## Authors' contributions

JCAT - leading of development of the study conceptualisation, design, refining of protocol and write up for publication. LK - contribution to refining and substantial contribution to writing up of the protocol for publication. GHWO - major input into study conceptualisation, design and protocol publication. EMM - contribution to development of study design, statistical issues, contribution to protocol publication. JB/TT - expert respiratory input contribution to study conceptualisation and refining on outcome measures, statistical issues and substantial contribution to writing up of the protocol for publication. TV/JWJL/AJP - contribution to study and intervention development and contribution to protocol publication.

All authors read and approved the final manuscript.

## Pre-publication history

The pre-publication history for this paper can be accessed here:

http://www.biomedcentral.com/1471-2466/9/52/prepub
